# How do Policy and Institutional Settings Shape Opportunities for Community-Based Primary Health Care? A Comparison of Ontario, Québec and New Zealand

**DOI:** 10.5334/ijic.2514

**Published:** 2017-06-27

**Authors:** Tim Tenbensel, Fiona Miller, Mylaine Breton, Yves Couturier, Frances Morton-Chang, Toni Ashton, Nicolette Sheridan, Alexandra Peckham, A Paul Williams, Tim Kenealy, Walter Wodchis

**Affiliations:** 1 University of Auckland, NZ; 2 University of Toronto, CA; 3 University of Sherbrooke, CA

**Keywords:** community-based primary health care, Canada, New Zealand, policy, institutions

## Abstract

Community-based primary health care describes a model of service provision that is oriented to the population health needs and wants of service users and communities, and has particular relevance to supporting the growing proportion of the population with multiple chronic conditions. Internationally, aspirations for community-based primary health care have stimulated local initiatives and influenced the design of policy solutions. However, the ways in which these ideas and influences find their way into policy and practice is strongly mediated by policy settings and institutional legacies of particular jurisdictions. This paper seeks to compare the key institutional and policy features of Ontario, Québec and New Zealand that shape the ‘space available’ for models of community-based primary health care to take root and develop. Our analysis suggests that two key conditions are the integration of relevant health and social sector organisations, and the range of policy levers that are available and used by governments. New Zealand has the most favourable conditions, and Ontario the least favourable. All jurisdictions, however, share a crucial barrier, namely the ‘barbed-wire fence’ that separates funding of medical and ‘non-medical’ primary care services, and the clear interests primary care doctors have in maintaining this fence. Moves in the direction of system-wide community-based primary health care require a gradual dismantling of this fence.

## Introduction

Community-based primary health care describes a model of service provision that is oriented to the population health needs and wants of service users and communities, and has particular relevance to supporting the growing proportion of the population with multiple chronic conditions. The ideal of community-based primary health care incorporates an understanding of primary health care which places an emphasis on the principles of equity, community partnership while addressing social determinants of health [1,2]. Community-based primary health care also involves a realignment of health system resourcing, emphasising the need for more ‘upstream’ approaches to prevention and care in the management of chronic conditions, and inter-professional approaches to service delivery. Thirdly, community-based primary health care recognises the need for action outside the health sector to implement health agendas and inter-sectoral approaches to health [3].

Internationally, aspirations for community-based primary health care have stimulated local initiatives and influenced the design of policy solutions. However, the ways in which these ideas and influences find their way into policy and practice is strongly mediated by policy settings and institutional legacies of particular jurisdictions.

Both the Canadian Institutes of Health Research and the New Zealand Health Research Council identified the importance of building cross-jurisdictional policy-relevant research regarding community-based primary health care A resulting collaboration between researchers in New Zealand and the two most populous provinces in Canada (Québec and Ontario) Implementing Integrated Care For Older Adults With Complex Health Needs (iCOACH) is examining the implementation of local integrated care initiatives considering policy, organizational, provider and patient and informal carer perspectives.

In this article, we outline the key policy and institutional features of New Zealand and the Canadian provinces of Ontario and Québec that shape the opportunities for community-based primary health care to emerge and take root. These jurisdictions share a number of fundamental similarities in the design of political and health sector institutions. Nevertheless, each jurisdiction has followed a distinct path. We begin with a high-level account of the key institutional features of Canadian and New Zealand health policy. This is followed by a more detailed account at the jurisdictional level which covers the organisational landscape pertinent to health and social care; the range of service delivery models; the specific mechanisms that are used to integrate the activities of different organisations and sectors; and important policy developments over the past 15–20 years.

We then compare these jurisdictions in terms of three factors that facilitate and/or impede the emergence of community-based primary health care: the extent of inter-organisational integration; the availability and use of policy levers; and the practices of policy implementation. This is a preliminary step in the iCOACH research collaboration.

## Comparing Canada and New Zealand

Canada and New Zealand share a range of health system and population health characteristics. Both countries ensure that their citizens have access to an extensive range of publicly-funded health care services, and both countries fund the bulk of these services from taxation revenue. Both countries also have aging populations, an increasing burden of chronic conditions and rising health care costs. Significant disparities in health outcomes [4] between indigenous and non-indigenous populations mean that older indigenous peoples are considerably more likely to suffer from multiple, chronic conditions at an earlier age.

Both countries have Westminster-based systems of parliamentary democracy, a product of their settlement by European colonists. Another legacy of this is the shared experience of the indigenous peoples of Canada and New Zealand of colonization, and the denial of their sovereignty by the colonisers [5]. Health inequalities such as those noted above are a product of these colonial and postcolonial histories. In both countries, treaties between colonisers and indigenous peoples have shaped constitutional arrangements that in turn shape health policy. In New Zealand, the Treaty of Waitangi signed in 1840 establishes a partnership of equals, with the implication that equity of determinants and outcomes of health are firmly embedded in health policy and practice [6]. By contrast, in Canada, treaty and land claims arrangements vary widely across the country, with many issues unresolved. Māori comprise over 15% of New Zealand’s population, compared to 4.3% for Aboriginal people in Canada (First Nations, Inuit, Metis) [7]. Another legacy of European settlement in Canada is the presence of two national languages, French and English, and a distinct culture and identity in the province of Québec.

Table 1 compares the key features of health care financing. The proportion of Gross Domestic Product spent on health is broadly similar in Canada and New Zealand; however New Zealand’s Gross Domestic Product per capita is significantly lower, accounting for its lower level of health spending per capita in monetary terms. In both countries, public sources are by far the largest contributors to the financing of health care. While both countries have private insurance industries, New Zealand’s performs a ‘substitute’ role for the public system covering niches such as elective surgery that are publicly funded, but where insurance provides faster access. In contrast to New Zealand, Canadian private insurance is a ‘supplement’ covering health services that are not publicly funded.

**Table 1:** Key Features of Health Care Financing in Canada and New Zealand.

	Canada	New Zealand

Health care spending as a % of Gross Domestic Product (2013) [8]	10.2%	9.5%
Health care spending per capita (US $ Purchasing power parity 2013) [8]	4351	3328
Percentage of total health expenditure from public sources (2013) [8]	70.6%	79.8%
Percentage of total health expenditure from out-of-pocket payments (2013) [8]	14.3%	12.6%
Percentage of total health expenditure from private insurance	12.8% [9]	5.0% [10]

Despite these broad similarities, the health systems of Canada and New Zealand differ substantially in key respects. The first key difference concerns the mix of services covered by government funding. In Canada, while hospital services and primary medical care are provided at no cost to patients, the costs of ‘non-medical’ services and pharmaceutical items are largely borne by service users, either directly, or through private insurance. New Zealand has more extensive public coverage of pharmaceutical costs, but many patients incur significant co-payments in primary medical care.

The second key difference is constitutional. Canada is a federation, whereas New Zealand is a unitary state. Under Canada’s federal structure of 10 provinces and three territories, health services and delivery are predominantly provincial responsibilities, although the federal government does provide additional insured benefits and health services to specific populations, and financially supports the delivery of health care by provinces and territories.

The key process shaping Canada’s institutional landscape was the development of Medicare in the 1950s and 1960s, codified at the federal level in the Canada Health Act of 1984. Medicare is widely seen as a defining characteristic of Canadian identity, with cost-free access for “medically necessary” doctor and hospital services as a core tenet, reinforced by a largely effective ban against a parallel system of privately financed or insured hospital and physician services. However, rather than constituting a unified health care delivery system, Medicare is a system through which the federal government contributes funding to the provinces and territories under the terms and conditions of the Canada Health Act. As well, the Canada Health Act’s focus on physician and hospital services leaves many essential services – including outpatient pharmaceuticals, rehabilitation services, and home and community care – as discretionary additions to provincial or territorial health insurance plans, which vary widely across the country. While heavily constrained within the core basket of Medicare goods and services, private finance and private insurance play a key role in the domains beyond physician and hospital services.

These arrangements are the enduring legacy of Medicare’s underlying “historical bargain”, forged in the early 1960’s in Saskatchewan as a response to that province’s introduction of Canada’s first publicly-funded health care insurance system. Essentially, this bargain was that the medical profession ceded the government’s right to fund hospital and doctor care, while the government ceded the profession’s right to control the organization and delivery of medical care [11]. While this initial bargain did not preclude physicians from opting out of the health insurance plan or charging patients in excess of what the plan paid, the subsequent federal Canada Health Act of 1984, pushed the provinces to eliminate physician “extra billing” and hospital user fees.

While similar issues and debates were instrumental in shaping New Zealand’s health system institutions, the institutional legacy differs in key respects. In New Zealand, public hospital services and many community-based services for citizens are provided at no cost to those who use them [12,13]. However, the high public share of health expenditure disguises high levels of out-of-pocket payments for primary care consultations for people aged 13 and over. This feature can be traced back to New Zealand’s “historical bargain” between the state and the medical profession in the late 1930s over the right to charge co-payments in primary medical care. New Zealand was one of the first countries that attempted to introduce universal coverage for health care as one part of the Social Security Act of 1938. While hospital medical professionals effectively agreed to become state employees (while retaining the right to practice privately as well), doctors working in primary practice successfully held out for the right to charge co-payments [14,15].

However, public spending accounts for a far greater share of funding for community-based care, long-term care and pharmaceuticals compared to Canada. Due to a highly effective public, co-ordinated purchasing arrangement that keeps prices down, access to pharmaceuticals is heavily publicly subsidized, while expenditure on pharmaceuticals is among the lowest in the Organization for Economic Cooperation and Development [16]. Prescription items dispensed outside of the hospital setting incur a NZ$5 patient co-payment, those dispensed by hospitals or specialists cost patients NZ$15. Some home care and disability (community care) support is publicly funded but means-tested as are government subsidies for residential aged care services. New Zealand also has a separate, ‘no-fault’ accident insurance scheme – the Accident Compensation Corporation – which is funded through taxation and employer contributions and accounts for about 8% of health spending [17]. Medical and rehabilitation costs related to accidents and injuries are covered by this scheme. Taken together, these factors mean that a wider range of health and social services are covered by the public purse in New Zealand than Canada.

However, a more fine-grained comparison of institutional and policy settings in Ontario, Québec and New Zealand is required. Under the overarching model of Canadian Medicare outlined above, within the federation there is considerable diversity, such that the most important factors that affect community-based primary health care, including the range of services funded publicly, the organisational landscape, models of service delivery; mechanisms of integration; and policy settings) are each determined at the provincial rather than the national level. We describe these features in greater detail in the following section.

## Jurisdictional Features – Ontario, Québec and New Zealand

### Ontario

#### Organisational Landscape

Ontario’s Ministry of Health and Long Term Care provides overall strategic direction and leadership for the health system. Despite government attempts during the late 1990s to rationalize the hospital system, which resulted in the forced closure of some smaller hospitals, and the amalgamation of others, Ontario hospitals remain private, albeit mostly not-for-profit entities with their own independent boards. Efforts to link hospital funding more closely to activity and quality, including beyond the hospital (e.g., activity-based funding reflecting the volume of surgical services provided), have increased in recent years, and are slowly supplementing global funding based on historical patterns.

Another more recent attempt at system reform to deal with fragmentation, silos, and disjointed service across the care continuum occurred in 2007 when 14 Local Health Integration Networks were introduced across the province of Ontario and assumed responsibility for local health system planning and community engagement. They are also responsible for funding a wide range of health service providers and sectors such as hospitals, long-term care homes, community support service providers, community health centres, Community Care Access Centres, psychiatric facilities, and mental health and addiction service providers, as well as managing the majority of service agreements with health service providers [18].

#### Service models

In principle, community-based primary health care spans health and social care sectors, including primary medical care, home care and non-medical community support services, covering a range of regulated and non-regulated providers. In practice, however, these sectors and providers remain siloed in Ontario.

Since the 1980s, Ontario has implemented successive waves of primary care reform, producing a plethora of reform models, each with widely varying organizational characteristics, focus and “spread” [19]. For the most part, these approaches to reform have focused mostly on the funding of medical services provided by primary care physicians, with an intention to move beyond traditional fee-for-service models. For example, two main reform models, Family Health Groups and Family Health Organizations, require no more than three physician owners (not necessarily co-located), paid via “enhanced” fee-for-service or mixed fee-for-service/capitation, with financial incentives for specified disease prevention and management procedures (e.g., cancer screening or diabetes management).

Other, but less widespread models, have attempted to change the organization of primary care practice. For example, physician-led multidisciplinary models such as Family Heath Teams require co-location with additional funding to support interdisciplinary teams of providers and expanded medical services. However, Family Heath Teams provide care for less than 20% of the population. Another important service model in Ontario is Community Health Centres which feature salaried physicians, interdisciplinary teams, community boards, and a concern with health promotion and population health. While moving toward a more expansive definition of community-based primary health care, Community Health Centres are limited by policy fiat to serving underserviced and marginalized populations such as the poor, recent immigrants, and persons with special needs such as persons with disabilities.

In spite of provincial efforts to promote these primary care reform models, in 2012, approximately a quarter (26%) of Ontario’s Family Physicians remained in fee-for-service solo practice. More than half (53%) were in “groups” such as Family Heath Groups paid through alternative funding mechanisms (such as blended fee for service and capitation); with less than a fifth working in multidisciplinary practices such as Family Health Teams, Community Health Centres or Aboriginal Health Access Centres. Nurse Practitioners lead some primary care practices and are employed in many Family Health Teams and Community Health Centres.

In the arena of home care, formal home care services such as nursing or rehabilitation therapy have been provided through Ontario’s 14 publicly-funded, regionally-based Community-Care Access Centres. These centres do not charge user fees, but nor do they guarantee that eligible individuals will receive needed services, as there is no universal entitlement to home care. Even medically-necessary services such as nursing may or may not be accessible. This depends on home care budgets, which vary substantially on a per capita basis across the province, and eligibility criteria, which increasingly emphasize very high clinical needs. As such, people with lower needs (and their caregivers) often need to find care alternatives. Adding another layer of complication, Community Care Access Centres do not directly provide services; rather they purchase services from not-for-profit and for-profit providers.

Non-medical community supports may be accessed through a constellation of mostly smaller-scale, local, not-for-profit, volunteer-driven Community Support Services Agencies. Community Support Services Agencies vary widely in terms of access, service capacity and eligibility and they tend to be “thin on the ground” outside major urban areas. All Community Support Services Agencies focus on the delivery of non-medical supports for Instrumental Activities of Daily Living, such as meals and transportation; some offer a single service (such as meals-on-wheels), while others offer a more extensive range of services. Like Community Care Access Centres, Community Support Services Agencies provide no service guarantee; unlike Community Care Access Centres, they are required to charge user fees as a condition of Ministry of Health funding (usually on a sliding scale geared to income) creating a perverse economic incentive toward “free” hospital and doctor care.

#### Integrating Mechanisms

Although Ontario would seem to have in place all the elements of a broad continuum of primary health care, these different elements continue to operate relatively independently with no overall coordinating strategy and few mechanisms to integrate client care across providers and settings.

In the 1990s a market model was introduced in the home and community care sector. While contractual discipline provides some opportunity for funders to plan more integrated services, the experience of competitive contracting typically leads to fragmentation rather than integration [20].

In contrast to the home and community care sector, contractual mechanisms are not possible at the core of Medicare. In Ontario, physician payments flow directly from the provincial health insurance plan, and there are few restrictions on where or how they practice. Neither Local Health Integration Networks nor hospitals directly control physician funding; physician services are specifically exempted from Local Health Integration Network authority. The terms and conditions of primary care reform models are negotiated by the province and the Ontario Medical Association, a powerful interest which controls professional fee schedules and it retains an effective veto over reform of medical practice, including reform of primary care. These factors place considerable constraints on the scope for service integration.

While under Local Health Integration Network authority, Community Care Access Centres, Community Support Services Agencies, Long Term Care Homes and hospitals continue to operate under their own independent governance structures. Additionally, there are no formal mechanisms beyond referral to link primary care doctors to a broader constellation of community-based programs and services often required by individuals with multiple chronic needs and their unpaid informal caregivers.

To further complicate this landscape, many of the programs, services and providers which might be included under a more inclusive definition of community-based primary health care are located beyond the Ministry of Health and Long-Term Care and the Local Health Integration Networks. For example, housing (including community housing for older persons and persons with disabilities) falls largely under the auspices of the provincial Ministry of Municipal Affairs and Housing. Social assistance for low income individuals and support for persons with developmental disabilities fall mostly under the aegis of the provincial Ministry of Community and Social Services. Moreover, many community support services agencies in Ontario also draw on federal and municipal resources, including employment and immigrant settlement services.

#### Policy Developments

Efforts to push primary care reform remain constrained by “framework agreements” between the province and the Ontario Medical Association. Under these agreements, negotiated on a 4-year cycle, government may use its spending power to “incent” individual physicians to participate in primary care reforms such as interdisciplinary group practice, but funding for this may not be diverted from the negotiated physician payment pool, making the costs of such reforms effectively “add-ons”. Local Health Integration Networks have little ability to shape primary care models since the large majority of doctors continue to be paid directly by the provincial health insurance plan. This has meant that primary care reform “models” implemented in Ontario over the past two decades have focused mostly on payment, particularly the introduction of alternatives to fee-for-service (e.g., “blends” of fee for service and capitation), rather than on the establishment of interdisciplinary, community-based practices which span health and social care. Furthermore, the province, under a constrained budget and a constrained system, recently announced that it would not support the creation of additional interdisciplinary Family Heath Teams at this point in time.

Nevertheless, there is growing agreement that more extensive reform of primary care specifically, and care systems generally, is needed. For example, the Primary Health Care Expert Advisory Committee, established in 2013, drafted a report suggesting radical change for Ontario’s Primary Health Care system. The recent Price Report (2015) suggested a redesign of Ontario’s primary care sector based on a population-based model of integrated Primary Health Care delivery. This model would involve patient care groups which would be accountable to the Ministry of Health and Long-term Care through the Local Health Integration Networks.

Similar themes are reflected in Ontario’s recently-released *Road Map for Home and Community Care* 2015) [21] which calls for structural changes in the delivery of home care and community support services. For example, the Road Map has prompted new legislation (Patients First Act, 2016), which gave notice that provincially-funded home care agencies (Community Care Access Centres) would be subsumed under Ontario’s Local Health Integration Networks. As a result Local Health Integration Networks are now poised to become direct service providers rather than just funders. However, reflecting the influence of organized pressure groups, this legislation exempts doctors, hospitals and long-term care homes from Local Health Integration Network authority to issue operational directives.

Finally, as an attempt to address this disconnect between health and social services, the government of Ontario introduced ‘Health Links’, an initiative which has both medical and social needs based providers come together to think about how to work together to fix problems and mobilize delivery of coordinated care across the continuum of care for those with chronic complex conditions [22]. However, under the Patients First Act, Health Links appear likely to be superseded by new sub-regions which will take on most or all of this local “linking” role.

### Québec

#### Organisational Models

In Québec, the Ministry of Health and Social Services is the provincial authority responsible for health and social services in the province [23]. The healthcare system is based on single public governance organizations covering a large geographic territory and a broad continuum of social and medical services under the same governance, with the exception of a majority of family physicians working in community settings.

Based on 20 years of structural work around integrated care, recent organisational mergers have created larger regional organizations with more centralized decision making processes. Since 2015, based on a full integration model, the core organisations are the Integrated Health and Social Services Centres. Integrated Health and Social Services Centres were formed by merging almost all the public institutions providing health and social care, including youth centres and rehabilitation centres (for intellectual or physical disability and addictions), within their combined areas of jurisdiction. The 22 Integrated Health and Social Services Centers answer directly to the Ministry, and are responsible for planning and delivering services, including negotiating agreements with providers, community organizations and private sector resources. This major reorganization of the health and social service structure was introduced with the objectives of simplifying the patient trajectory, improving the circulation of information, and increasing savings by the reduction of management positions [24].

This configuration built on an earlier (2004) large-scale reorganization of Québec’s healthcare structure, where a variety of local service provision organisations, including Local Community Health Centers that deliver home care services and primary health care, long term care services and, in most of the cases, an acute care hospital were merged into a single structure called Health and Social Services Centres. At that time, 94 Health and Social Service Centres were created across the province at local level with a ‘population-based responsibility’ mandate to improve the health and well-being of a geographically defined population [25,26]. The objective has been to empower the reorganization of services adapted to the needs of the population living in a geographical area. Health and Social Services centers receive funding to deliver broad range of services, from preventive to more curative and palliative care.

#### Service models

Strong primary care delivery is an important component of health system performance [27]. However, Québec’s health and social services system developed historically without the presence of primary medical care. Until recently, delivery of primary medical care was at the periphery of the system rather than at its core [28] and has been focused on family physicians. Although nearly all family physicians are individually remunerated by public funds, most primary care practices are private enterprises, neither owned nor governed by the state. Historically, private medical practice in the community has been the dominant type of primary care in Québec, but these have established very few links with public healthcare organizations. The responsibility for organizing primary healthcare services was left to community-based private medical practices owned by a physician or a group of physicians.

However, Québec was among the first jurisdictions in the world in developing a model of Primary Health Care based on health and social services. Starting in the early 1970s, the government launched an ambitious reform project by creating Local Community Health Centres. Those organisations were merged into Health and Social Services Centers in 2004. Local Community Health Centers were entirely public, in terms not only of funding, infrastructure and resources, but also of governance. This model is based on interdisciplinary team such as physicians, nurses, occupational therapists, physiotherapists, nutritionists, psychologists and social workers. Local Community Health Centers provide both preventive and curative services, as well as support services such as home care. These more comprehensive primary health care organizations were among the first around the world of integrating social and medical services and delivering home care services. They are still an important component for delivering home care and social services but they are not the predominant model of primary care. Originally, Local Community Health Centers were meant to be the main entry point into the healthcare system and being the unique model of primary healthcare in Québec [29]. However, physicians’ associations vehemently opposed the practice conditions associated, particularly the fact that Local Community Health Centers physicians were salaried. Few family physicians (20%) have elected to practice in these facilities and only a small proportion of the population identifies them as their source of primary care services [30,31].

Given the Local Community Health Centers’ relative failure to attract enough physicians in this multidisciplinary model of primary healthcare, the Clair Commission in the year 2000 proposed a new primary care model, the Family Medicine Group. Since 2002, Family Medicine Groups, much like Family Health Teams in Ontario, consist of 6 to 10 physicians working in close collaboration with nurses in providing services to enrolled patients on a non-geographic basis (between 10,000 to 15,000 people per family medicine group) [32]. No new infrastructure was required for the majority of Family Medicine Groups as they were created from existing private practices. Since the Family Medicine Group policy was inaugurated in 2002, the number of accredited practices has been steadily increasing. In May 2016, there were 272 accredited Family Medicine Groups in the province, enrolling approximately 50% of the province’s population. The Family Medicine Group reform made provision for the recruitment of nurses and administrative staff, and the acquisition of information technology. Social workers and other professionals are being integrated into Family Medicine Groups. The vision is to build a stronger primary healthcare based on multidisciplinary teams. Those professionals will be transferred from Local Community Health Centers to collaborate more closely with family physicians in primary healthcare for complex patients. This is viewed as a way to facilitate collaboration among public structures and the predominantly privately-owned primary medical care providers.

In 2010, the Québec government announced it would support the role of nurse practitioners and fund the integration of 500 nurse practitioners into primary care teams over the next decade. This may lead to a redefinition of professional roles of nurses and family physician [33]. The vision is to develop primary health care practice based on interdisciplinary teams to deliver care to enrolled patients.

#### Integration Mechanisms

Under the 2004 and 2015 structural reforms, based on horizontal and vertical integration of several establishments under the same governance, the integrated organizations were mandated to develop collaborative or contractual arrangements with other providers (private and for non-profit) inside their territory that offered services needed by the population (e.g. community pharmacies, community organizations and medical clinics) as well as supra-regional entities such as tertiary care hospitals out of their areas of jurisdiction. They were mandated to lead the development of integrated and coordinated care for various groups of the population such as older adults and users of mental health services. The creation of these health services networks was based on a broad continuum of care under a unique governance structure to develop partnerships with other organizations such as medical practice in the community. But structural integration of several components of continuum of care does not mean the services are well integrated.

The Ministry of Health and Social Services also has many small contractual agreements with Family Medicine Groups. Funding for nurse practitioners, for example, can be accessed this way when family physicians enrol higher numbers of older patients and deliver services for complex patients in a broader scope. Integrated Centres for Health and Social Services also contract with non-government (third sector) organisations that provide home care services, and many of these organisations are very dependent on Integrated Centre for Health and Social Services contracts.

The local service networks provide another mechanism for negotiated agreements between Integrated Centres for Health and Social Services and providers such as non-government organisations and community pharmacists. However, the potential for this mechanism of integration has been undermined by the recent amalgamation of health regions because these arrangements require smaller geographic areas to work.

#### Policy Developments

Over the last decade, several changes were made by governments to improve primary health care in the community, based on patient-centred medical home components [34]. The Family Medicine Group model appears to be better accepted by the physicians than the Local Community Health Centers were, but it could be a way to softly privatise a part of the public system. This wider acceptance may be due to the model’s greater compatibility with the values of the medical profession, but more specifically because a fee-for-service remuneration system is maintained. In addition, the majority of Family Medicine Groups required no new infrastructure since they were created from existing private practices. In Québec, access to after-hours primary care has also been a high level policy priority over the past 20 years. Both the development of Family Medicine Groups and of Integrated Centres for Health and Social Services have been seen by government as ways to address this problem, but it remains an issue because family doctors have resisted efforts to increase access to after-hours care.

In February 2016, the Québec government passed a controversial law, Bill 20, aimed at addressing problems of access to family physicians. This law is seen as a strategy to negotiate with the doctor’s union to increase the proportion of patients attached to family doctors and for patients to have timely access to family doctors. Only 72% of the population are enrolled with a family doctor in the community. Family physicians have until January 2018 to improve enrolment of the population to 85%. If they do not achieve this objective, an income reduction penalty of 30% will apply. This is one of the first times a coercive strategy has been used as a way of changing doctor practice. While Bill 20 focuses on family doctors, the strategy to reach this goal is largely geared to encourage the development interdisciplinary teams to respond to patient needs in primary healthcare.

### New Zealand

#### Organisational Landscape

The New Zealand Public Health and Disability Act (2000) provides the underpinning legislative framework of New Zealand’s health and disability system. The Ministry of Health, primarily has a policy advice, development and monitoring role, and is the locus of public accountability. The Ministry also directly funds some services, including disability services for people aged under 65. The bulk of public expenditure on health and disability services (76%) is channelled through 20 District Health Boards according to a risk-adjusted population-based funding formula [16]. Under the New Zealand Public Health and Disability Act, District Health Boards are responsible for the planning of services for their local population, and for the implementation of national policy directives. District Health Boards provide some services directly, including hospitals, outpatient specialist services, community-based mental health, disability assessment and public health services and are purchasers of other services from for-profit and non-profit non-government organisations. These services include primary care, mental health and disability support services, home care services and residential aged care.

District Health Boards are governed by a combination of seven elected and up to four government-appointed members. Democratic governance of publicly provided health services has a history dating back to the 1870s [35]. However, the signals from central government are far clearer and stronger than signals from local citizens [36]. Public hospitals are bulk-funded, based upon estimated volumes and costs. New Zealand also has a small private hospitals sector catering mainly for elective surgery, and funded by private insurance and out-of-pocket payments [13].

Primary Health Organisations are non-government organisations that contract with primary care practitioners. There are currently 32 Primary Health Organisations, with an average enrolment of about 150,000 patients [37]. Almost all New Zealanders are enrolled with a general practice provider contracted to a Primary Health Organisation. Primary Health Organisations receive a population-based capitation formula for their enrolled patients, weighted according to the age, gender, rurality, and ‘high-user health card’ status [38]. Most of this public funding is passed on to contracted general practice based on their patient enrolment, but some funding streams are managed at Primary Health Organisation level for organisational infrastructure and management, and for service development initiatives. Funding for one separate stream – services to improve access- is weighted according to socio-economic deprivation and Māori and Pacific ethnicity [38].

#### Service Models

Most primary care is provided by family doctors and nurses working in a general practice team. Some services may also include community-based nurses, specialists and allied health professionals. Family doctors in New Zealand are referred to as general practitioners and registered nurses working in general practice are referred to as practice nurses. Group practices are the norm, and larger practices are becoming an important presence in response to policy initiatives and changes to the organisational environment. Only 6% of general practices are solo practitioners [39]. General practice services are predominantly organised around 10–15 minute consultations [16].

General practices receive a mix of capitation and fee-for-service payments. Since 2003, most of the public funding for primary care services has been paid through a population-based capitation formula. However, fee-for-service remains a significant component of primary care remuneration, with out-of-pocket fee for service payments comprising between 30 and 35% of general practice income [40]. In addition, the Accident Compensation Corporation reimburses primary care costs for accident-related injuries on a fee-for-service basis.

Provision of after-hours primary care is a contractual requirement for general practices and after hours arrangements vary between districts. In some areas it is effectively organised, formally or informally, by networks of general practices. In other areas, there is a sizeable for-profit presence which has not been connected to other primary care providers [41,42].

Since the 1990s, Māori and Pacific Island health service providers have become a significant presence in community-based primary health care. These organisations provide a broad range of social and health services, and their practice is informed by principles of ‘whānau ora’ (loosely translated as family health) which has at its core a philosophy of integrating services around the specific needs and circumstances of extended family groups. Only a few of these Māori and Pacific providers directly employ family doctors, but they do employ a range of practitioners including primary care nurses, dental, social workers, as well as many workers that are part of the ‘unregulated’ health workforce.

Most social care services are provided by non-government organizations and funded by District Health Boards (for people aged 65 and over). Social care services for those aged under 65 are funded directly by the Ministry of Health with payments being made in a range of different ways including personal budgets/vouchers which have gradually been introduced since the 1990s [12].

#### Integration Mechanisms

Governance of publicly-funded services provided by non-government organisations is handled through formal contracts between providers and District Health Boards or the Ministry. For primary care, there is a national contract (the Primary Health Organisation Services Agreement). Under this agreement Primary Health Organisations are accountable for meeting the national health targets pertaining to primary care. For other community-based services, contracts are typically short-term (1–2 years) and commercially contestable (in theory, if not always in practice). These formal structures are strongly shaped by New Zealand’s adoption of widespread New Public Management reforms between 1987 and 1996 built almost exclusively on the foundations of principal-agent theory [43,44]. When contracting was first introduced in the 1990s, a traditional, competitive approach was taken, with contracts being short-term and contestable. However, since 2013 District Health Boards and Primary Health Organisations are required to have an Alliance Agreement in place (alongside their Primary Health Organisation Services Agreement) [45]. This is intended to stimulate an “alliance contracting” framework in which purchasers and providers work collaboratively to achieve the desired outcomes. Nevertheless, contestability and short-term contractualism remain the standard template for relationships.

#### Policy developments

Between 1983 and 2001, New Zealand experienced three rounds of major restructuring of the health system. An initial round of reform in the 1980s consolidated and merged small hospital boards into larger entities. A radical restructure in the 1990s instigated a strict separation of the functions of purchasing and providing health care. The 2001 restructure which created District Health Boards sought to reintegrate the purchasing and provision of health services after a seven-year experiment with organisational separation. The turn of the millennium also saw the turn to an explicit, population-based approach to health policy and funding.

Primary health care was also high on New Zealand’s health policy agenda around 2000. At this time, government prioritised problems of access and equity in primary care – many of which were seen as stemming from the high levels of family doctor co-payments. Inspired by the Alma Ata primary health care agenda, the Primary Health Care Strategy successfully introduced a new organisational format for primary care in the form of Primary Health Organisations. This built on developments within the Independent Practitioner Association movement of the 1990s. At this time, a niche emerged for community governed non-profit primary health care organisations that employed family doctors on salary as part of inter-professional teams [46]. Although not created by government, these organisations (including Māori and Pacific providers referred to above), provided the template for the government’s vision of Primary Health Organisations, and many became Primary Health Organisations in the mid-2000s. The Primary Health Care Strategy also shifted the public funding of general practice services from fee-for-service reimbursement to capitation for an enrolled population.

Primary Health Organisations were originally intended to be organisations that brought together multiple professions, that were governed (at least in part) by community representatives, and were expected to use some of their funding for planning to address population health needs through primary care [47]. Around the same time as the introduction of the Primary Health Care Strategy, regulatory changes also provided significant scope for registered nurses and nurse practitioners to play a larger role in primary care, but the growth and uptake of nurse practitioner roles in primary care has been very slow [48].

However, the implementation of the Primary Health Care Strategy reflected a light touch by government [49]. General practice providers were the first professional group to contract with Primary Health Organisations to establish an enrolled population but other professional groups such as community pharmacy and midwives have remained independent and their funding is separate to Primary Health Organisation funding.

Most of the new investment in primary care was in the form of increased patient subsidy to reduce the financial barrier to accessing primary care services. This was passed through to contracted general practices with few requirements other than to reduce patient fees by an agreed amount. Other PHO funding streams were available for high need populations but many were reluctant or unable to develop specific initiatives to address unmet need and inequities. The community governance requirement was resisted by many family doctors and was eventually watered-down significantly. Since 2008, the smaller community-governed primary care providers have struggled to maintain financial viability and have largely disappeared as government policy promoted Primary Health Organisation mergers and consolidation [50]. Many Māori health providers that were once Primary Health Organisations have now become part of ‘mainstream’ Primary Health Organisations.

In the 2000s, the government also introduced new strategies for people with disabilities and older people [51,52]. Although signalling a prioritisation of these issues, these policies also suffered from being primarily aspirational, and being vague for implementers.

Since the late 2000s, government policy shifted to emphasise integration of services between community settings and hospitals, primarily through alliances between District Health Boards and Primary Health Organisations, and the closer alignment of their planning processes and performance measures [53].

However, arguably the most significant policy development in the past ten years regarding integration and community-based primary health care has been the introduction of Whānau Ora in 2010 [54]. Based on the central *wh*ā*nau ora* philosophy, this has seen an extra injection of funds (about NZ$130 m per year) to support Māori and Pacific providers to further develop integrated or ‘wrap-around’ social services and support for extended family groups [54]. Many, but not all, of these providers are part of Primary Health Organisations. This policy development opens up some new possibilities for more comprehensive community-based primary health care, although a clear picture of its impact on the health and social service environment is yet to emerge.

### Comparison of institutional and policy environments

Based on the above descriptions of the key features of institutional and policy environments of the three jurisdictions, we now look to compare them in terms of the opportunities and constraints for better system integration in order to promote community-based primary health care.

### Organisational integration

The first dimension of comparison is the organisational landscape, and whether it is aggregated or disaggregated. On this dimension, Ontario sits at one end of the spectrum with its highly disaggregated structure of Local Health Integration Networks, independent hospitals, multiple organisational and funding models of primary care, (until recently) a separate structure of home care (Community Care Access Centres) and a diverse range of non-government organizations. There are few linkage mechanisms regarding medical services, although contractual mechanisms are extensive in social and long term care [20]. The capacity of Local Health Integration Networks to achieve more integrated, community-based primary health care has been limited by the fact that primary care physicians are exempted from their control, and budgets for hospitals and Community Care Access Centres continue to be determined at the provincial level largely on the basis of historical funding patterns.

New Zealand is at the other end of the spectrum with the large majority of funding for both health and social care services being channelled through the District Health Boards as the purchasers of health and social care services at a district level. Furthermore, most health and social care service providers are part of the two key health sector organisations – District Health Boards and Primary Health Organisations. Primary Health Organisations provide for the aggregation of primary care, and this has important system implications for governance. In addition, relationships between District Health Boards and Primary Health Organisations are mediated by formal contracts and alliancing arrangements. Some important players such as Māori providers that do not employ doctors are less integrated into the system, although they too have extensive contractual relationships with District Health Boards and Primary Health Organisations. New Zealand policy in recent years has also emphasised the gradual formalisation of collaborative network structures between District Health Boards and Primary Health Organisations.

Québec sits close to New Zealand on this spectrum with state organisational structures which include most or all relevant providers *except* primary medical care. There are some mechanisms (at the margins) for contractual linkage between Integrated Centre for Health and Social Services (and their predecessors) and primary care organisations (Family Medicine Groups).

Of course, this integration-supporting architecture does not necessarily imply movement to a broader notion of community-based primary health care. New Zealand’s arrangements and developments since the late 2000s have arguably strengthened the focus of Primary Health Organisations on primary *medical* care, and, in structural terms, community-based primary health care has lost much of the foothold that it had prior to 2009. Similarly, in Québec, the Local Community Health Centres that were the key organisational locus of community-based primary healthcare have merged into larger organisational entities since 2015. In contrast, key structural locations for community-based primary health care remain in Ontario (Community Health Centres).

### Policy levers

A second dimension of comparison is the strength of policy levers. Again, the three jurisdictions are spread across a similar spectrum. Arguably, New Zealand has the strongest policy levers. In the health sector, there are no ongoing, formalised processes in which the government is obliged to negotiate with organised medicine regarding policy initiatives [55]. The introduction and consolidation of Primary Health Organisations are attributable to state capacity and New Zealand governments have also strongly shaped the linkage mechanisms between health sector organisations.

In New Zealand, the constitutional status of the Treaty of Waitangi offers one lever that has been used to address inequities which is not available to Canada. This is reflected in the activities and intentions (if not always the outcomes) of mechanisms such as the Health Equity Assessment Tool, the obligation of District Health Boards and Primary Health Organisations to prepare joint Māori health plans, as most significantly in the development and implementation of Whānau Ora as a policy priority. The introduction of Whānau Ora also represents in the most high profile of a number of experiments with inter-sectoral funding of services, although it remains to be seen whether such mechanisms can counteract the problems of ‘siloed’ funding.

Both New Zealand and Québec have also made concerted attempts to incorporate population health objectives into their health policy settings [37]. Compared to Ontario (and other Canadian provinces), Québec has a stronger etatist system and culture which has resulted in a system in which provincial government both funds and provides the bulk of health and social care services. Québec governments have been very active in restructuring the state-controlled health sector. However, like Ontario, there have been few effective policy levers for governing primary care. The recent attempt to set enrolment targets to deal with primary care access problems may be a pivotal test of the capacity of Québec’s government to steer.

Ontario has the weakest levers as is evidenced by the predominance of incremental primary care policy initiatives for the past thirty years [56]. However, in common with Québec, Ontario governments have considerably more scope to shape the policy and the institutional environment for non-medical services.

### Implementation

While some jurisdictions may have more powerful policy levers and institutional architecture to call upon than others, the effectiveness of such levers is dependent on what happens in implementation. Here the differences between New Zealand, Québec and Ontario are less marked.

New Zealand’s policy initiatives regarding community-based primary health care have frequently foundered on the rocks of implementation. Primary care practices operating in a private, for-profit environment successfully resisted the push to guarantee significant community influence on Primary Health Organisation governance [57]. The intention of increased primary care funding in the early 2000s was to introduce a universal funding formula and reduce the financial barrier to accessing general practice services. The universal formula funding was passed through Primary Health Organisations to contracted general practices with few requirements other than to reduce patient fees by an agreed amount. But the other funding streams introduced at Primary Health Organisation level to support initiatives to enhance access for marginalised populations have been piecemeal and have not generally changed established patterns of service delivery. Apart from the increased role for practice nurses, the inter-professional component of the Primary Health Care Strategy has not been realised. As another example of implementation failure, there has not been successful integration of health and disability services in New Zealand in spite of aggregated funding and organisational responsibility [58].

Québec’s policy history shows how primary care doctors successfully resisted the introduction of Local Community Health Centres in the 1970s, but showed more enthusiasm for a later model of primary care organisation (Family Medicine Groups) that was far more in line with their interests as private practitioners. More generally, the reliance on restructuring the organisational landscape over the past fifteen years indicates a preference for ‘top-down’ styles of policy implementation in Québec that are also likely to experience a challenging implementation environment. Policy initiatives that do not involve legislative instruments, but require local interpretation, adaptation and inter-organisational collaboration, (e.g., case management, planning tools, local networks with non-government organizations), have proven challenging to implement.

Ontario’s attempts to incrementally expand the range and scope of primary care have also met with implementation obstacles. Thus far, primary care reform has relied on altering payment mechanisms for primary care doctors. One optimistic view is that primary care reform, while slow off the mark, has now “entered a period of potentially transformative change” [59], and that it has, in fact, actually “transformed” the local health care landscape [56]. Less optimistically it could be argued, that where change has occurred, it has been mostly local and mostly at the margins; in Ontario, and in other provinces, the primary care “mainstream” continues to be defined mostly in terms of medical services provided to individual patients by individual family doctors, most of whom continue to work as small entrepreneurs in independent practices with few linkages to other providers or sectors. In all jurisdictions, the success of reforms seems dependent on their acceptability to the medical profession.

In New Zealand and Ontario, short-term contracts and hierarchical performance measurement embedded in parts of the health system in the 1990s has often been a significant obstacle to integration and to community-based service provision. In both jurisdictions, contracts have been heavily focused on deliverable outputs and processes in competitive environments – an institutional arrangement that makes integration extremely challenging. These contractual disciplines generally do not affect family doctors, but are heavily focused on community-based providers. Accordingly, these providers are very vulnerable to changing funder priorities and budgetary pressures and face high transaction costs of contract renewal and compliance [46,20].

## Conclusion

In this overview, we have sought to provide some context about healthcare in Ontario, Québec and New Zealand and provide high-level comparisons of key institutional and policy features that shape the ‘space available’ for models of community-based primary health care to take root and develop. We should not forget that progress towards more integrated models of community-based health care involves many services, multiple organizations various professional groups and policy actors. Those key actors have to align their goals with greater health system goals and their individual needs. It is therefore a highly complex field of policy and governance.

Our analysis suggests that some jurisdictions have more favourable conditions than others to implement the integration of a continuum of health and social care. Where change has happened more readily, there appear to be two key conditions: i) a supporting policy framework or coordinating strategy to improve the connectivity, communication, and accountability across relevant health and social sector, providers, and settings and ii) a range of policy levers available and used by governments. On both conditions, the New Zealand policy environment appears to offer the largest scope, with Ontario’s environment significantly less conducive, and Québec situated in between. Nevertheless, in each jurisdiction there remain important structural and institutional barriers to implementation and diffusion of policies and/or local models that promote community-based primary health care.

The first crucial common barrier across all jurisdictions is that the opportunities for community-based primary health care are limited by arrangements in which funding for primary medical care dominates the landscape, and is separated from the funding of ‘non-medical’ care. This ‘barbed-wire fence’ is difficult, painful and potentially dangerous to traverse for individual organizations and governments alike. Yet as long as primary medical care is funded in different ways to the other components of community-based primary health care, the environment for an integrated, community-based approach to primary care will be unsupportive. This is illustrated most starkly in Québec, where there is an institutional divide between funding of primary medical care and all other health and social care services. In Ontario, medical and ‘non-medical’ primary health care are funded separately, with each of these domains subject to further funding fragmentation. In New Zealand, while Primary Health Organization may provide a possible space for funding integration across the medical/non-medical divide, the degree to which this divide is bridged in practice remains limited.

Secondly, in each jurisdiction, the success of any policy initiative moving in the direction of community-based primary health care – including changes to funding models – has been dependent on their acceptability to the medical profession. This is hardly surprising. In Canada, the corporatist policy processes in which provincial medical associations ensure the protection of primary medical care funding is entrenched by the Canada Health Act. Without this ringfencing, medical associations would be less able to effectively represent the financial and professional interests of their membership in circumstances in which they are almost totally dependent on the state as a source of remuneration. In New Zealand, there is more scope (theoretically) for more integrated funding for community-based primary health care due to the presence of Primary Health Organisations, and their contractual relationships with government funding organisations. Yet here, the divide between funding primary medical care and broader health and social care has also become strongly institutionalised, and, by and large integrated funding for community-based primary health care represents add-ons to the core of medical, general practice funding.

Based on these findings, a key to progress is the role of medical professionals and the organisations that represent them. As long as, individually and collectively, they see their interests as threatened by more integrated approaches to funding, the prospects for progress through the route of explicit policy decisions and initiatives appear bleak as these will be resisted either during policy formulation and decision-making or during implementation.

In this context, one strategy to progress may be to solidify and gradually expand the range and scope of ‘niches’ in which more integrated funding does operate. Governments in each of the three jurisdictions have scope to make it easier for organisations and service models that already operate under more integrated funding models. Ontario has its Community Health Centres, Québec still has had its old model of community health and social services centres and New Zealand has had community-based PHOs in the past, and Whānau Ora providers currently. These models are limited (either explicitly or implicitly) to specific, usually marginalised populations and groups, and policy attempts to ‘mainstream’ these niche approaches have failed. However, governments can take active measures to enable these models such that they become more attractive to a wider range of providers in the health and social care environment.

A complementary strategy is for governments to actively define and measure public value outcomes such as improved access to health services, improved quality, health outcomes and equity of outcomes, and to reward such improvements. This strategy should be approached by small steps rather than ambitious policy designs. Crucially, the criteria used to evaluate the performance of providers must be meaningful to physicians while incorporating broader system objectives. Such an approach would also entail a wider range of providers recognising over time that the only way to improve outcomes is to participate in models in which the ‘barbed-wire fence’ between primary medical care funding and the other elements of integrated, community-based primary health care is dismantled. Together, the key is to foster environments in which more primary care doctors see it *as in their interests* to be part of more integrated funding environments.

Although our review is limited to three jurisdictions and two countries, it has wider implications because the barbed-wire fence that separates medical from non-medical primary care can be found in most high-income countries. Our future research in the iCOACH project will include a strong focus on whether and how this fence can be traversed in organisational practice and jurisdictional policy, with a view to providing a research base that can foster the growth of community-based primary health care.
